# Mitochondrial fission is required for thermogenesis in brown adipose tissue

**DOI:** 10.1371/journal.pone.0312352

**Published:** 2024-12-09

**Authors:** Yuta Ibayashi, Nao Hasuzawa, Seiji Nomura, Masaharu Kabashima, Ayako Nagayama, Shimpei Iwata, Miyuki Kitamura, Kenji Ashida, Yoshinori Moriyama, Ken Yamamoto, Masatoshi Nomura, Lixiang Wang

**Affiliations:** 1 Department of Medicine and Bioregulatory Science, Graduate School of Medical Science, Kyushu University, Maidashi, Higashi-ku, Fukuoka, Japan; 2 Division of endocrine and metabolism, Department of Internal medicine, Kurume University School of Medicine, Kurume, Japan; 3 Department of Medical Biochemistry, Kurume University School of Medicine, Kurume, Japan; Zhejiang University of Technology, CHINA

## Abstract

Brown adipose tissue (BAT) thermogenesis is pivotal for maintaining body temperature and energy balance. Mitochondrial morphology is dynamically controlled by a balance between fusion and fission, which is crucial for cell differentiation, response to metabolic insults, and heat production. Dynamin-related protein 1 (Drp1) is a key regulator of mitochondrial fission. This study investigates the role of Drp1 in BAT development and thermogenesis by generating Drp1-deficient mice. These mice were created by crossing Drp1 floxed mice with fatty acid-binding protein 4-Cre (*aP2*-Cre) transgenic mice, resulting in *aP2*-Cre^+/-^*Drp1*^flox/flox^ (*aP2-Drp1*^f/f^) mice. The *aP2-Drp1*^f/f^ mice exhibited severe BAT and brain hypoplasia, with the majority dying within 48 hours postnatally, highlighting Drp1’s crucial role in neonatal survival. Impaired thermogenic responses were observed in *aP2-Drp1*^f/f^ mice, characterized by significantly decreased expression of thermogenic and lipogenic genes in BAT. Ultrastructural analysis revealed disrupted mitochondrial morphology and reduced lipid droplet content in BAT. The few surviving adult *aP2-Drp1*^f/f^ mice also showed impaired BAT and brain development, along with BAT thermogenesis dysfunction during cold exposure. Our findings underscore the essential role of Drp1-mediated mitochondrial fission in BAT thermogenesis and neonatal survival, providing insights into potential therapeutic approaches for metabolic disorders.

## Introduction

Adipose tissue is a crucial endocrine organ, playing a significant role in regulating energy metabolism through the control of insulin sensitivity and appetite. It comprises two distinct types: brown adipose tissue (BAT) and white adipose tissue (WAT). BAT is characterized by its high mitochondria content, which enables it to generate heat by burning excess sugar during cold exposure, a process known as thermogenesis [1–3].

Mitochondria morphology is dynamically regulated in response to cellular energy requirements, differentiation processes, and pathological conditions [4, 5]. Mitochondrial dynamics are determined by a balance between fusion and fission, which is regulated by fusion factors, such as mitofusin 1/2 (Mfn1/2), optic atrophy 1 (Opa1), and fission factor dynamin-related protein 1 (Drp1) [6–8]. Mfn2 has been identified as essential for BAT thermogenic function, with Mfn2 deficiency leading to significant BAT dysfunction, impaired respiratory capacity, and a reduced response to adrenergic stimulation [9, 10].

Previous research has demonstrated that Drp1 is more abundantly expressed in BAT than in WAT, with its levels increasing during brown adipocyte differentiation. Chronic exercise also elevates Drp1 protein expression in BAT, indicating that mitochondrial fission plays a role in the metabolic adaptation of BAT to chronic exercise [11]. The inhibition of Drp1 using mdivi-1 has been shown to impare beige adipocyte differentiation and associated mitochondrial biogenesis [12]. In the inguinal WAT of cold-exposed mice, ellagic acid induces beige remodeling by regulating mitochondrial dynamics. This browning effect is abolished by the Drp1 inhibitor, mdivi-1 [13]. Moreover, dietary fat overload reprograms mitochondrial dynamics in brown fat, marked by reduced Drp1 levels, leading to a failure to activate the thermogenic program during cold exposure in both mothers and their offspring [14].

Drp1-mediated mitochondrial fission is necessary for norepinephrine-induced thermogenic activation in BAT and controlling energy expenditure. Inhibition of Drp1 using a dominant-negative mutant decreases the ability of brown adipocytes to induce mitochondrial uncoupling upon activation [15]. Human studies using multipotent adipose-derived stem cells have shown that beige adipocyte mitochondria exhibit enhanced oxidative capacity and sustained fission driven by Drp1. Additionally, cold exposure has been found to promote the recruitment of the peroxisomal protein to mitochondria, enhancing protein kinase A-mediated phosphorylation of Drp1, which in turn increases uncoupled respiration and thermogenesis in BAT [16].

Acknowledging foundational prior work has provided specific insights into the mechanistic role of Drp1 in regulating mitochondrial fission within BAT and its critical importance for thermogenesis. To further explore the critical role of mitochondrial fission in BAT growth and thermogenesis, and to advance our understanding by detailing the consequences of Drp1 deficiency, we generated mice lacking Drp1 in BAT by crossing *Drp1* floxed mice with fatty acid binding protein 4-Cre (also known as *aP2*-Cre) mice. This study aims to elucidate the specific effects of Drp1 deficiency on BAT growth and thermogenesis and its broader implications, particularly focusing on its relevance to neonatal survival.

## Materials and methods

### Animals

Fabp4-Cre (*aP2*-Cre) mice, which express the Cre recombinase gene under the control of the *Fabp4* gene promoter [B6. Cg-Tg (Fabp4-cre)1Rev/j], were obtained from Jackson Laboratory. These mice express CRE abundantly in brown and white adipose tissue and only marginally in other tissues, such as skeletal muscle, liver, and heart, as judged by northern blot analysis and a functional test using *R26R–ROSA* mice [17, 18]. *Drp1*^flox/flox^ mice, in which exons 2 and 3 of the *Drp1* gene are flanked by loxP sites by homologous recombination, were generated as previously described [19]. a*P2-Drp1*^f/f^ mice were generated by crossing *Drp1*^flox/flox^ mice with Fabp4-Cre mice. The mice were genotyped for the *Drp1* conditional allele and the Cre transgene using PCR primers as described [19].

#### Methods of sacrifice

Mice were sacrificed using a humane method to minimize suffering. Specifically, euthanasia was performed by carbon dioxide (CO2) inhalation, followed by cervical dislocation to ensure death. CO2 inhalation is recognized as a humane and effective method of euthanasia for rodents.

#### Methods of anesthesia

Prior to any surgical procedure, mice were anesthetized using isoflurane inhalation anesthesia. This method ensures rapid induction and recovery, allowing precise control over the depth of anesthesia.

#### Efforts to alleviate suffering

All animal experiments were performed in accordance with the Guide for the Care and Use of Laboratory Animals and were approved by the Ethics Committees on Animal Experimentation of Kyushu University Graduate School of Medicine (A27-218-0). Mice were housed at 22°C-24°C, if not otherwise indicated, they were provided with soft bedding and had free access to food (normal chow diet; 5.4% fat, CRF-1; Orient Yeast, Tokyo, Japan) and water under a 12 h/12 h light/dark cycle. Samples from adult mice were collected following anesthesia with isoflurane. Any signs of discomfort were addressed promptly, and animals showing persistent distress were euthanized humanely.

#### Hotplate treatment for pups

Infant mice immediately after birth were raised on a hot plate (already warmed to 35°C), together with their mothers, and observed as the hotplate treatment group.

#### Acute cold exposure for adult mice

The body temperature of the animals acclimated to room temperature 26°C, first measured with a rectal probe (RET-3, Physitemp Instruments, Inc., Clifton, NJ). Immediately afterwards, the mice were transferred to 4°C. Their body temperature was then measured every 30 min throughout the experiment until their temperature fell below 10°C. They were provided free access to water, but feeding was prohibited during the test.

### Western blot analysis

Protein samples from the newborn mice were taken within 12 h after birth. Samples of the adult mice were taken between 10:00 AM and 12:00 AM. Fresh tissues were homogenized, and protein content was measured using a BCA Protein Assay kit (Thermo Scientific, Rockford, IL, USA). The samples were then mixed with Laemmli sample buffer (1:1; Bio-Rad, Hercules, CA, USA) and heated for 5 min at 95°C. We performed sodium dodecyl sulfate polyacrylamide gel electrophoresis (SDS-PAGE) on equal amounts of protein from each sample, followed by transfer to a polyvinylidene difluoride (PVDF) membrane, and incubated with primary antibodies, followed by horseradish peroxidase (HRP)-linked secondary antibodies. S1 Table lists the primary and secondary antibodies used. Protein bands were visualized using the ECL Western Blotting Detection System (GE Healthcare, Buckinghamshire, UK). The signals were quantified using Image J software (NIH, Bethesda, MD, USA). Uncropped western blot images are shown in S1 Fig.

### Total mRNA isolation and real-time PCR

Total RNA was isolated using TRIzol reagent (Invitrogen Corporation, Carlsbad, CA, USA) according to the manufacturer’s instructions. For real-time-PCR assays, we converted 1000 ng of total RNA into first-strand complementary DNA (cDNA) using the QuantiTect Reverse Transcription Kit (QIAGEN, Hilden, Germany) according to the manufacturer’s instructions. Next, we used the cDNA for quantitative real-time PCR using 2× SYBR Green PCR Master Mix and monitored the process with an ABI Prism 7500 sequence detection system (Thermo Fisher Scientific, Rockford, IL, USA). S2 Table lists the primer sequences of the selected genes. We normalized relative gene expression versus to that of *Gapdh* expression.

### Histological analysis

For histochemical and immunohistochemical analyses, adipose tissue and several organ tissues were fixed and embedded in paraffin or OCT compound using standard methods. For H&E staining, we stained the paraffin sections with Mayer’s hematoxylin solution for 5 min, followed by counterstaining with 0.5% (v/v) eosin alcohol solution for 2 min. For immunostaining, we diluted primary and secondary antibodies in Can Get Signal^™^ Immunoreaction Enhancer Solutions A and B, respectively (Toyobo, Osaka, Japan). S1 Table lists the primary and secondary antibodies used. Sections were analyzed using a BZ-8000 microscope (Keyence, Osaka, Japan). Apoptotic cells were detected by TdT-mediated dUTP nick end labeling (TUNEL) staining using an Apoptag kit (Millipore, Darmstadt, German).

### Electron microscopic analysis

For chemical fixation of animal tissues for electron microscopy, random newborn mice samples were taken within 12 h after birth at room temperature. The mice were decapitated and exsanguinated, then iBAT were immediately cut into small blocks and fixed with 2% paraformaldehyde (PFA) and 2% glutaraldehyde (GA) in 0.1 M phosphate buffer (PB), pH 7.4, at 4°C overnight. The samples were dehydrated and embedded in Epon812. The grids were observed by a transmission electron microscope (JEM-1400Plus; JEOL Ltd., Tokyo, Japan) at an acceleration voltage of 80 kV. Digital images (3296 × 2472 pixels) were captured with a CCD camera (EM-14830RUBY2; JEOL Ltd., Tokyo, Japan).

### Statistical analysis

Data are expressed as means ± SEMs. All data were analyzed using GraphPad Prism software (GraphPad Software Inc., La Jolla, CA, USA). Unpaired t-test, two-way ANOVA, and log-lank test were used as appropriate. A P-value <0.05 was considered statistically significant.

## Results

### Postnatal death of *aP2- Drp1*^f/f^ mice

**Fig 1:**
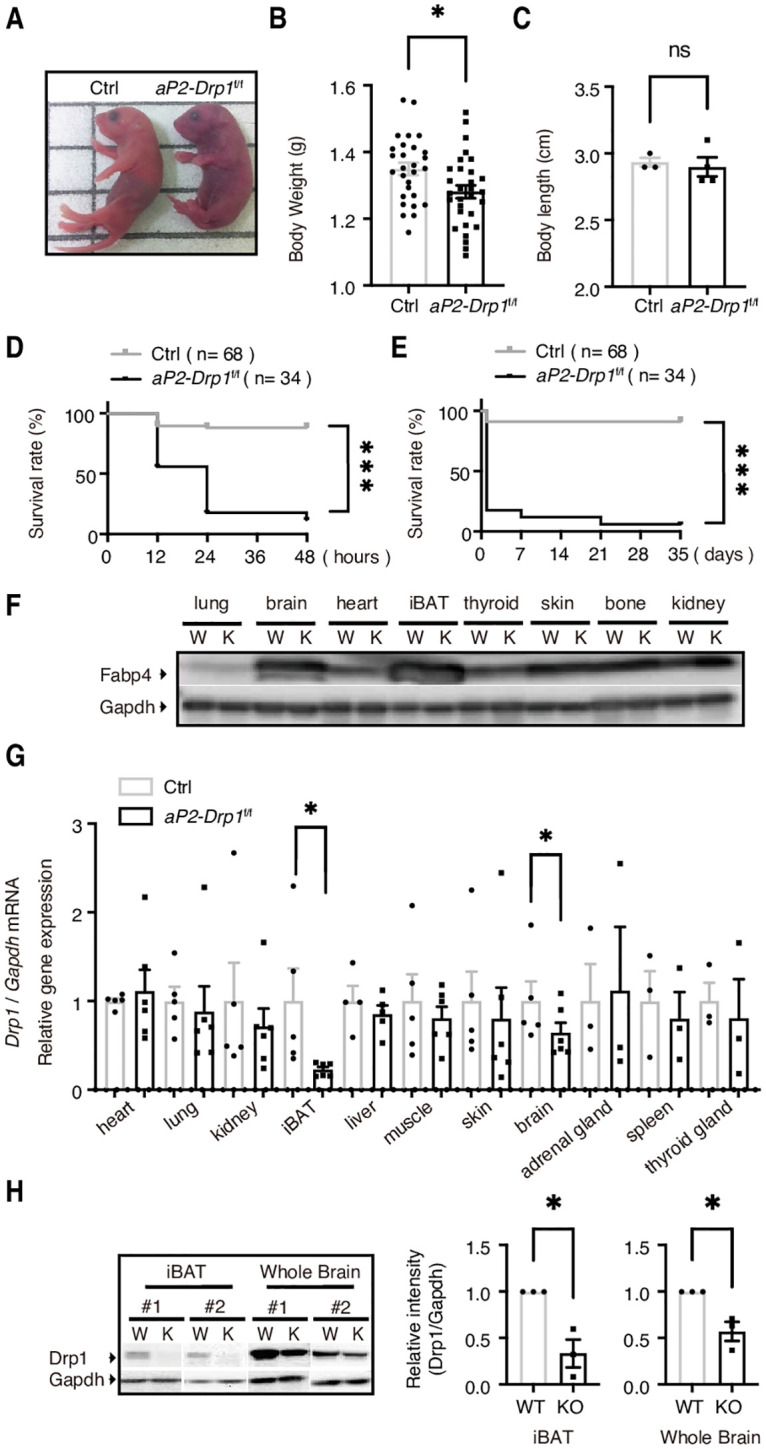
Postnatal death of *aP2-Drp1*^f/f^ neonatal mice. A. *aP2-Drp1*^f/f^ mice immediately after birth (0 h) compared with wild-type littermate controls. B. Body weight from control and *aP2-Drp1*^f/f^ mice at 0 hours after birth was measured and presented as means ± SEM. *P < 0.05 determined by an unpaired *t*-test. (n = 28–30). C. Body length from the nose to tail base from control and *aP2-Drp1*^f/f^ mice at 0 hours after birth were measured and presented as means ± SEM. (n = 3–4). D-E, Signs of life were closely observed to assess survival and were presented as Kaplan-Meier survival curves. Between-group difference was tested by the log-rank test. ***P < 0.001. F. Tissues were harvested from control and *aP2-Drp1*^f/f^ mice within 12 hours after birth and subjected to SDS-PAGE and western blot analysis. G. Gene expression profiles are shown for the heart, lung, kidney, iBAT, liver, muscle, skin, brain, spleen, adrenal gland, and thyroid gland from control and *aP2-Drp1*^f/f^ mice within 12 hours after birth. Data were shown as means ± SEM. * P < 0.05 determined by an unpaired *t*-test. (n = 3–6). H. Western blot analysis was performed to evaluate the expression of Drp1 in the iBAT and whole brain from control and *aP2-Drp1*^f/f^ mice within 12 hours after birth. Gapdh was used as a loading control. The biological duplicate samples in each condition numbered as #1 and #2. Results are expressed as means ± SEM. The results of quantitative analysis are shown in the right panel. *P < 0.05 determined by an unpaired *t*-test. (n = 3). Abbreviations: Drp1; dynamin-related protein 1, K; knock-out, W; wild-type, Gapdh; glyceraldehyde 3-phosphate dehydrogenase, iBAT; interscapular brown adipose tissue.

Genotype analysis of littermates from intercrosses between *aP2-Drp1*^f/+^ (*aP2*-Cre^+/-^*Drp1*^flox/+^) and *Drp1*^flox/flox^ demonstrated that genotype frequencies followed Mendelian inheritance patterns on embryonic day 18.5 (E18.5) and postnatal day 0 (P0: 0 h after birth). However, when young mice were marked with ear-tags and their tail tips were cut for genotyping, *aP2-Drp1*^f/f^ mice were rarely observed by the 21st day after birth (Table 1). These results indicate that most *aP2-Drp1*^f/f^ mice died after birth and barely survived beyond 3 weeks. Interestingly, all surviving mice were female, raising intriguing questions about how biological sex influences metabolic processes, including thermogenic activity, in response to Drp1 knockout and its effects on postnatal survival. As shown in Fig 1A, *aP2-Drp1*^f/f^ mice exhibited no gross abnormalities compared to *Drp1*^f/f^ (referred to as control) mice at 0 h after birth. More detailed observations of newborn *aP2-Drp1*^f/f^ mice revealed highly restricted movements. They moved clumsily, breathed with difficulty (prone to cyanosis), and were unable to suckle normally. The body weight of *aP2-Drp1*^f/f^ mice at 0 h after birth was decreased (Fig 1B), whereas body length showed no difference between *aP2-Drp1*^f/f^ and control mice (Fig 1C). Postnatal death occurred in most of the *aP2-Drp1*^f/f^ mice, with 90.5% dying prematurely within 48 hours after birth, and over 86% of deaths occurring within the first 24 hours (Fig 1D). Only a few *aP2-Drp1*^f/f^ mice survived beyond the first few days, and nearly all of them (97.6%) died within 3 weeks after birth (Fig 1E).

**Table 1:** Number of *aP2-Drp1*^f/f^ (KO: Fabp4 Cre^+/-^-*Drp1*^flox/flox^), *aP2-Drp1*^f/+^ (Hetero: Fabp4 Cre^+/-^-*Drp1*^flox/+^), *Drp1*^f/f^, and *Drp1*^f/+^ mice at E18.5, P0, and P21 from the intercrosses between Hetero and *Drp1*^f/f^ mice.

Birth of day	Genotype	Number of mice	Percentage (%)
E18.5	*aP2-Drp1* ^f/f^	20	21
	*aP2-Drp1* ^f/+^	25	26
	*Drp1* ^f/f^	23	23
	*Drp1* ^f/+^	29	30
	Total	97	100
P0	*aP2-Drp1* ^f/f^	22	28
	*aP2-Drp1* ^f/+^	20	26
	*Drp1* ^f/f^	19	24
	*Drp1* ^f/+^	17	22
	Total	78	100
P21	*aP2-Drp1* ^f/f^	9	2.4
	*aP2-Drp1* ^f/+^	133	36
	*Drp1* ^f/f^	122	33
	*Drp1* ^f/+^	110	29
	Total	374	100

### Drp1 deletion in *aP2-Drp1*^f/f^ mouse BAT and brain

A series of studies were consecutively performed to determine the potential cause of postnatal death in *aP2-Drp1*^f/f^ mice. To determine the specific effect of *Drp1* gene deletion in tissues expressing Fabp4, the tissue expression of Drp1 and Fabp4 was measured. Fabp4 expression was present in a wide range of tissues. Relatively high expression was found in brain and interscapular brown adipose tissues (iBAT). Fabp4 is also expressed in skin, bone, and kidney. In addition, Fabp4 was weakly, but to some extent detected, in other organs including the lung, heart and thyroid gland of neonatal mice (Fig 1F). Real time PCR analysis revealed an approximate 70% and 40% reduction of *Drp1* mRNA levels in iBAT and brain in *aP2-Drp1*^f/f^ neonatal mice, respectively (Fig 1G). Similarly, western blot analysis revealed that the relative amount of Drp1 was significantly decreased in iBAT and brain of *aP2-Drp1*^f/f^ neonatal mice compared with control mice (Fig 1H). The absolute weights of the brain and kidney in *aP2-Drp1*^f/f^ neonatal mice were reduced compared to those in control mice. However, no significant differences were observed in the absolute and relative weights of other organs, including the liver, spleen, heart, and lung, between the two groups of mice (Table 2).

**Table 2:** Comparison of absolute (mg) and relative (mg/g body weight) organ weights between newborn control and *aP2-Drp1*^f/f^ mice.

			Control	*aP2-Drp1* ^f/f^	P ^a^
Organ weight ^b^,^c^	brain	Absolute	79.73±3.74	64.43±5.10	< 0.05
		Relative	53.80±1.65	47.55±2.89	NS
	liver	Absolute	69.90±1.11	60.56±3.02	NS
		Relative	45.31±1.03	44.64±2.82	NS
	spleen	Absolute	2.42±0.18	2.20±0.25	NS
		Relative	1.63±0.10	1.62±0.16	NS
	kidney	Absolute	16.67±0.43	14.38±0.82	< 0.05
		Relative	11.27±0.21	10.66±0.49	NS
	heart	Absolute	8.67±0.23	7.98±0.64	NS
		Relative	5.86±0.11	5.93±0.49	NS
	lung	Absolute	40.03±0.88	37.55±1.43	NS
		Relative	27.15±0.96	27.99±1.51	NS

^a^, Statistical analysis was carried out by Student’ s t test, NS: not significant.

^b^, Newborn mice were examined, n = 5–6 per group, Values are means ± SEM.

^c^, Wet weights of paired organs were averaged for each mouse, and the single value was used to calculate mean ± SEM in each genotype.

### Hypoplasia of the iBAT and maladaptation to cold stress in *aP2-Drp1*^f/f^ mice

**Fig 2:**
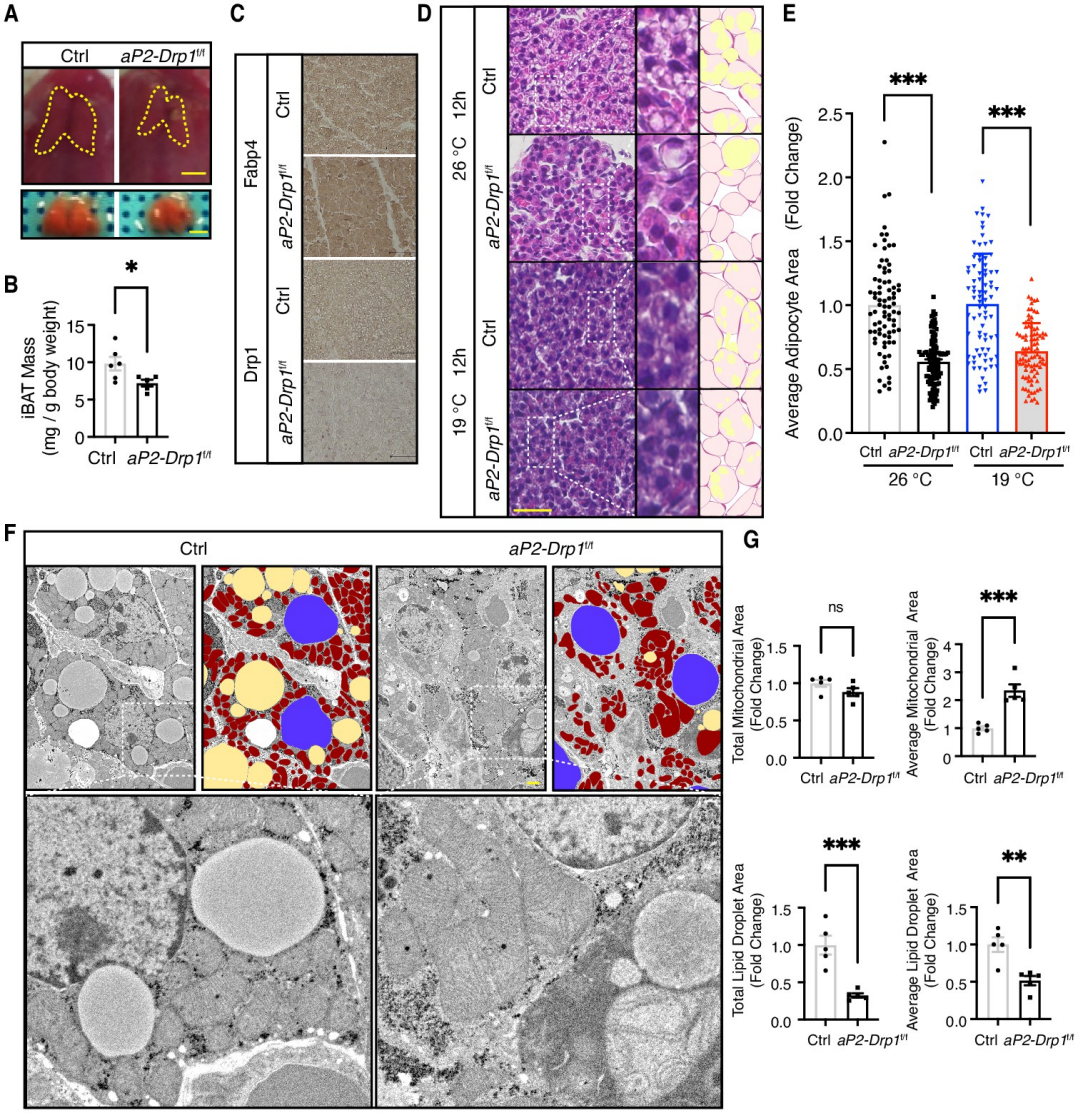
iBAT characterization of the *aP2-Drp1*^f/f^ neonatal mice. A-B. Morphology (A) and relative mass (B) of iBAT from control and *aP2-Drp1*^f/f^ mice at 0 h after birth. Yellow dashed lines track iBAT boundaries. Scale bar = 1.0 mm. n = 5–6, *p < 0.05 C. Immunohistological staining for Fabp4 and Drp1 in control and *aP2-Drp1*^f/f^ mice at 0 h after birth. Scale bar = 50 μm. D. H&E staining of control and *aP2-Drp1*^f/f^ mice iBAT sections. The mice were treated at room temperature (26°C) or cold temperature (19°C) at 12 h after birth. Areas indicated with white dashed squares are enlarged and shown in the middle. Traces of adipocytes (purple) and lipid droplets (yellow) are shown on the right. Scale bar = 600 μm. E. Quantification of the average area of the adipocytes. n = 80–109, ***p < 0.001 F. Transmission electron microscopy (TEM) of mitochondrial morphology in iBAT from control and *aP2- Drp1*^f/f^ mice at room temperature. False color TEM micrograph showing mitochondria (red), lipid droplets (yellow), and nucleus (blue). Areas indicated with white dashed squares are enlarged and shown on the lower panel. Scale bar = 1μm. G. Quantification of the total area and the average area of the mitochondria and lipid droplets. n = 5, **p < 0.01, and ***p < 0.001 determined by an unpaired *t*-test. Abbreviations: Drp1; dynamin-related protein 1, Fabp4; Fatty acid binding protein 4, HE; Hematoxylin and Eosin.

We next examined the histological changes in Fabp4-expressing tissues derived from neonatal control and *aP2-Drp1*^f/f^ mice. No significant histological alterations were observed in tissues including the heart, lung, skin, kidney, smooth muscle, and liver (S2 Fig). When the iBAT was dissected by removing the fat pad from between the shoulder blades, we found that iBAT from *aP2-Drp1*^f/f^ mice was smaller compared to control littermates (Fig 2A), and the relative mass of iBAT in *aP2-Drp1*^f/f^ mice was also reduced (Fig 2B). The protein expression levels of Drp1 and Fabp4 in iBAT were further assessed by immunohistochemistry. Fabp4 expression was observed in both *aP2-Drp1*^f/f^ and control mice. As expected, a remarkable decrease of Drp1 protein in the iBAT cells was observed in *aP2-Drp1*^f/f^ mice compared with control mice (Fig 2C). Further observations of hematoxylin and eosin staining also showed that *aP2- Drp1*^f/f^ brown adipocytes were smaller in size and contained fewer lipid-droplets compared with those in control mice at room temperature (26°C). Correspondingly, similar results were observed in *aP2-Drp1*^f/f^ mice under cold stress of 19°C for 12 hours after birth, which induces a robust thermogenic response based on previous preclinical experimental studies [20] (Fig 2D). Quantitative analysis revealed that in *aP2-Drp1*^f/f^ mice, the average size of adipocytes was significantly decreased both at room temperature and under cold stress conditions (Fig 2E). Ultrastructural analysis of iBAT at room temperature (26°C) showed robust architectural differences in mitochondria between control and *aP2-Drp1*^f/f^ mice (Fig 2F). In control mice, mitochondria had circular structures with intact cristae evenly distributed in the marginal part. In contrast, the mitochondria of *aP2-Drp1*^f/f^ mice were larger, branched, or bent with disrupted cristae structure. Furthermore, quantitative analysis revealed that in *aP2-Drp1*^f/f^ mice, mitochondrial size was significantly increased, while both the total area and the average size of lipid droplets were significantly decreased (Fig 2G).

### Drp1 defect affects thermogenic and lipogenic gene expression in iBAT of *aP2-Drp1*^f/f^ mice

**Fig 3:**
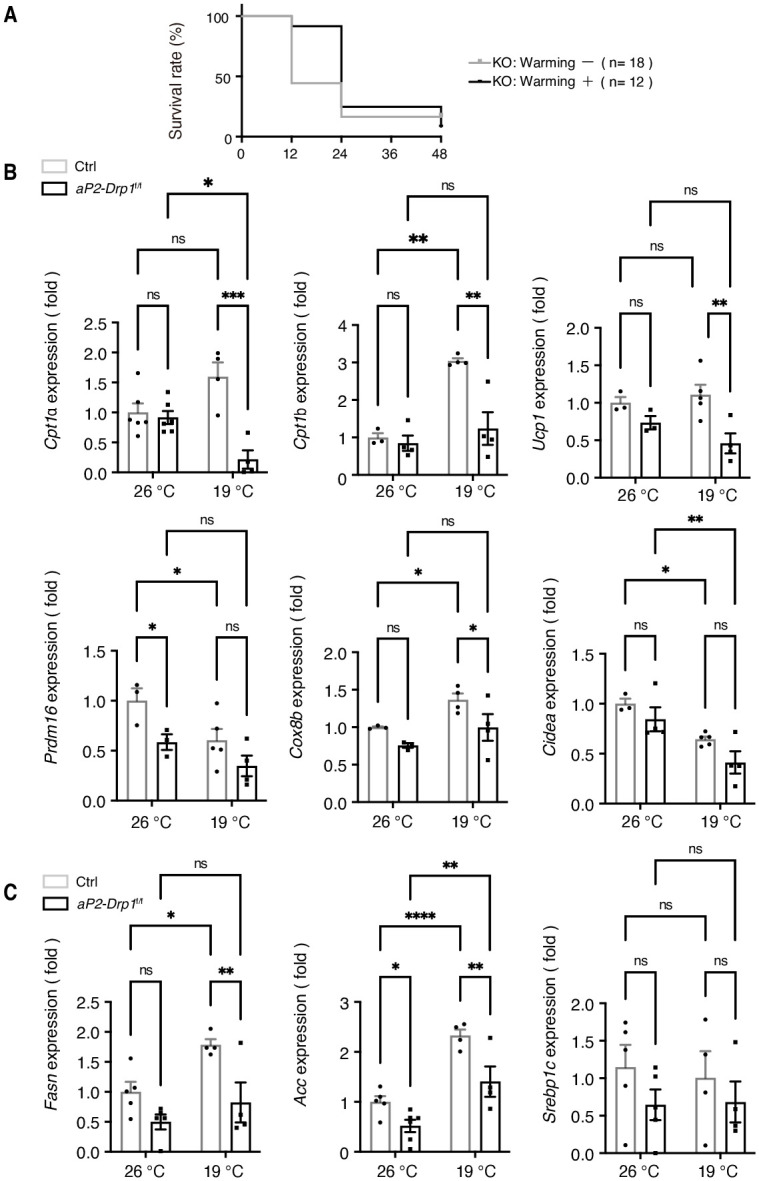
Reduced expression of thermogenic and lipogenic genes of *aP2-Drp1*^f/f^ neonatal mice. A. Rescuing effect of a hot plate (35°C) on the survival of *aP2- Drp1*^f/f^ mice relative to room temperature (26°C) at indicated time after birth. Signs of life were presented as Kaplan-Meier survival curves. Between-group difference was tested by the log-rank test. (n = 12–18). B-C. Thermogenic (B) and lipogenic (C) gene expression in iBAT from neonatal control and *aP2-Drp1*^f/f^ mice. Mice were chronically exposed at room temperature (26°C) or cold temperature (19°C), respectively, at 12 h after birth. Data are shown as means ± SEM; *p < 0.05, **p < 0.01, and ***p < 0.001 determined by two-way ANOVA with Bonferroni’s post hoc test. (n = 3–6). Abbreviations: *Ucp1;* uncoupling protein 1, *Cpt1*; carnitine palmitoyl transferase 1, *Cox8b;* cytochrome c oxidase subunit 8B, *Prdm16;* PR domain containing 16, *Cidea*; cell death-inducing DNA fragmentation factor alpha-like effector A, *Fasn;* fatty acid synthase, *Acc;* acetyl CoA carboxylase, and *Srebp1c;* sterol regulatory element binding protein 1.

At birth, neonates must rapidly adapt from the warm intrauterine environment to a colder extrauterine environments. Since newborn hypothermia can be lethal, this adaptation is crucial [21, 22]. Next, we tried to rescue *aP2-Drp1*^f/f^ mice by increasing the environmental temperature to determine the mechanism of the Drp1 defect on the survival of *aP2-Drp1*^f/f^ mice. As shown in Fig 3A, administration of the hotplate (35°C) significantly increased the survival rate of *aP2-Drp1*^f/f^ mice at 12 hours after birth. However, hotplate administration did not allow most of the *aP2-Drp1*^f/f^ mice to survive beyond 24 hours after birth, suggesting that the low survival rate of *aP2-Drp1*^f/f^ mice may be due to other factors in addition to BAT dysfunction.

Above, hypothermia among newborns is considered an important contributor to neonatal mortality in *aP2-Drp1*^f/f^ mice, the expression of genes involved in thermogenesis and lipogenesis in iBAT was investigated both at room temperature (26°C) and under cold stress (19°C for 12 h after birth) conditions. Carnitine palmitoyl transferase 1 (Cpt1) imports fatty acids into the mitochondria and increases fatty acid oxidation. As shown in Fig 3B, the expression of *Cpt1a* showed an increased trend under colder environment in control mice, whereas *Cpt1a* was decreased in *aP2-Drp1*^f/f^ mice. The *Cpt1b* was increased in the colder environment in control mice, whereas *Cpt1b* was not changed at the low temperature in *aP2-Drp1*^f/f^ mice, suggesting defective fatty acid oxidation in *aP2-Drp1*^f/f^ mice. The expression of uncoupling protein 1(*Ucp1*) was decreased at the low temperature *aP2-Drp1*^f/f^ mice compared to control mice. Of note, the expression of the PR domain containing 16 (*Prdm16*) gene, which is the main transcriptional regulators in iBAT development was significantly decreased in *aP2-Drp1*^f/f^ mice at room temperature, and another thermogenic gene, cytochrome c oxidase subunit 8B (*Cox8b*), was decreased at low temperature. Molecular markers of brown-like adipocytes, which control the switch from WAT to BAT, such as cell death-inducing DNA fragmentation factor alpha-like effector A (*Cidea*) also showed a decreased trend in *aP2-Drp1*^f/f^ mice, although there was no statistically significant difference.

Next, we measured the expression levels of the de novo lipogenic genes, fatty acid synthase (*Fasn*), acetyl CoA carboxylase (*Acc*), and sterol regulatory element binding protein 1 (*Srebp1c*). The expression levels of *Fasn* and *Acc* was significantly increased after cold exposure in control mice, whereas they were decreased in *aP2-Drp1*^f/f^ mice both under room temperature and cold exposure conditions (Fig 3C).

### Hypoplasia in the brain of *aP2-Drp1*^f/f^ neonatal mice

**Fig 4:**
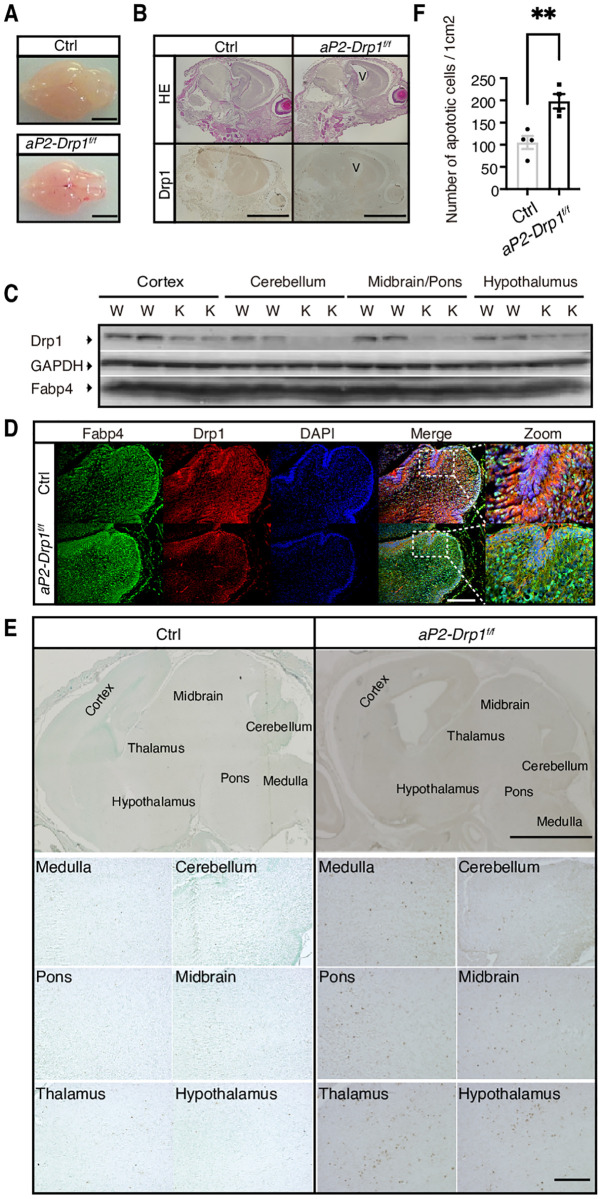
Brain disintegration in *aP2- Drp1*^f/f^ neonatal mice. A. The macroscopic view of the brains of the control and *aP2-Drp1*^f/f^ mice at 0 h after birth. Scale bar = 3.0 mm. B. Histological H&E staining (upper panel) and IHC staining for Drp1 (lower panel) of sagittal sections from control and *aP2-Drp1*^f/f^ brains at 0 h after birth. Scale bar = 3.0 mm. C. Western blotting was performed to evaluate the expression of Drp1 and Fabp4 in the following portions of the brain: cerebral cortex, cerebellum, midbrain, and hypothalamus. Gapdh was used as a loading control. D. Representative images of Fabp4 and Drp1 in the cerebellum of control and *aP2-Drp1*^f/f^ mice at 0 h after birth. Fabp4-positive cells using Alexa Fluor 488-conjugated anti-rabbit IgG (green) and Drp1-positive cells were visualized using Alexa Fluor 594-conjugated anti-mouse IgG (red) and the nuclei were stained with DAPI (blue). Areas indicated with white dashed squares are enlarged and shown on the right side of the picture. Scale bar = 10 μm. E. Brain sections from newborn mice were subjected to TUNEL staining and visualized with HRP-DAB (dark brown dots). Nuclei were stained with methyl green as a nuclear counterstain. Scale bar = 10 μm. F. The quantitative analysis of apoptotic cells in the whole brain of control and *aP2-Drp1*^f/f^ mice at 0 h after birth. n = 4, **p < 0.01 determined by an unpaired *t*-test. Abbreviations: Drp1; dynamin-related protein 1, V; lateral ventricles, Fabp4; Fatty acid binding protein 4, DAPI; 4′,6-diamidino-2-phenylindole, HE; Hematoxylin and Eosin, IHC; immunohistochemistry, TUNEL; terminal deoxynucleotidyl transferase dUTP nick end labeling.

Investigating the systemic effects of Drp1 deficiency beyond BAT is crucial for understanding its broader physiological impact. Given the high expression of Fabp4 in the mouse brain and the known crosstalk between brain and BAT in the context of thermogenesis, we focused on examining brain tissues from *aP2-Drp1*^f/f^ mice, where Drp1 expression was found to be significantly down-regulated compared to controls. The brain size of newborn *aP2-Drp1*^f/f^ mice were smaller compared with those of control mice (Fig 4A). Hematoxylin and eosin staining revealed hypoplasia of the brain with expanded lateral ventricles in *aP2-Drp1*^f/f^ mouse brain (Fig 4B, upper panel). Drp1 immunostaining showed a remarkable decrease in Drp1 protein expression in *aP2-Drp1*^f/f^ mice compared with control mice (Fig 4B, lower panel). Next, we analyzed the expression of Drp1 in each part of the brain by western blot analysis. As shown in Fig 4C, Drp1 was decreased in the cortex, cerebellum, midbrain/pons, and hypothalamus. Immunofluorescent staining revealed scattered expression of Fabp4 in cerebellum, and the expression of Drp1 was decreased but not completely deleted in the cerebellum (Fig 4D).

Neural cell specific Drp1 knockout mice die shortly after birth because of brain hypoplasia with apoptosis [19]. To investigate whether apoptosis was increased in *aP2-Drp1*^f/f^ mouse brain, TdT-mediated dUTP nick end labelling (TUNEL) staining was performed. The scattered distribution of apoptotic cells was disclosed in serial sections of the brain of *aP2-Drp1*^f/f^ mice (i.e., medulla, cerebellum, midbrain/pons, thalamus, and hypothalamus) (Fig 4E). There was a significant increase in the number of TUNEL-positive cells in the brain of *aP2-Drp1*^f/f^ mice (Fig 4F). Thus, down-regulation of Drp1 resulted in the obstruction of mitochondrial fission and neuronal cell disintegration, followed by brain hypoplasia, and expanded lateral ventricles.

### BAT dysfunction of adult *aP2-Drp1*^f/f^ mice

**Fig 5:**
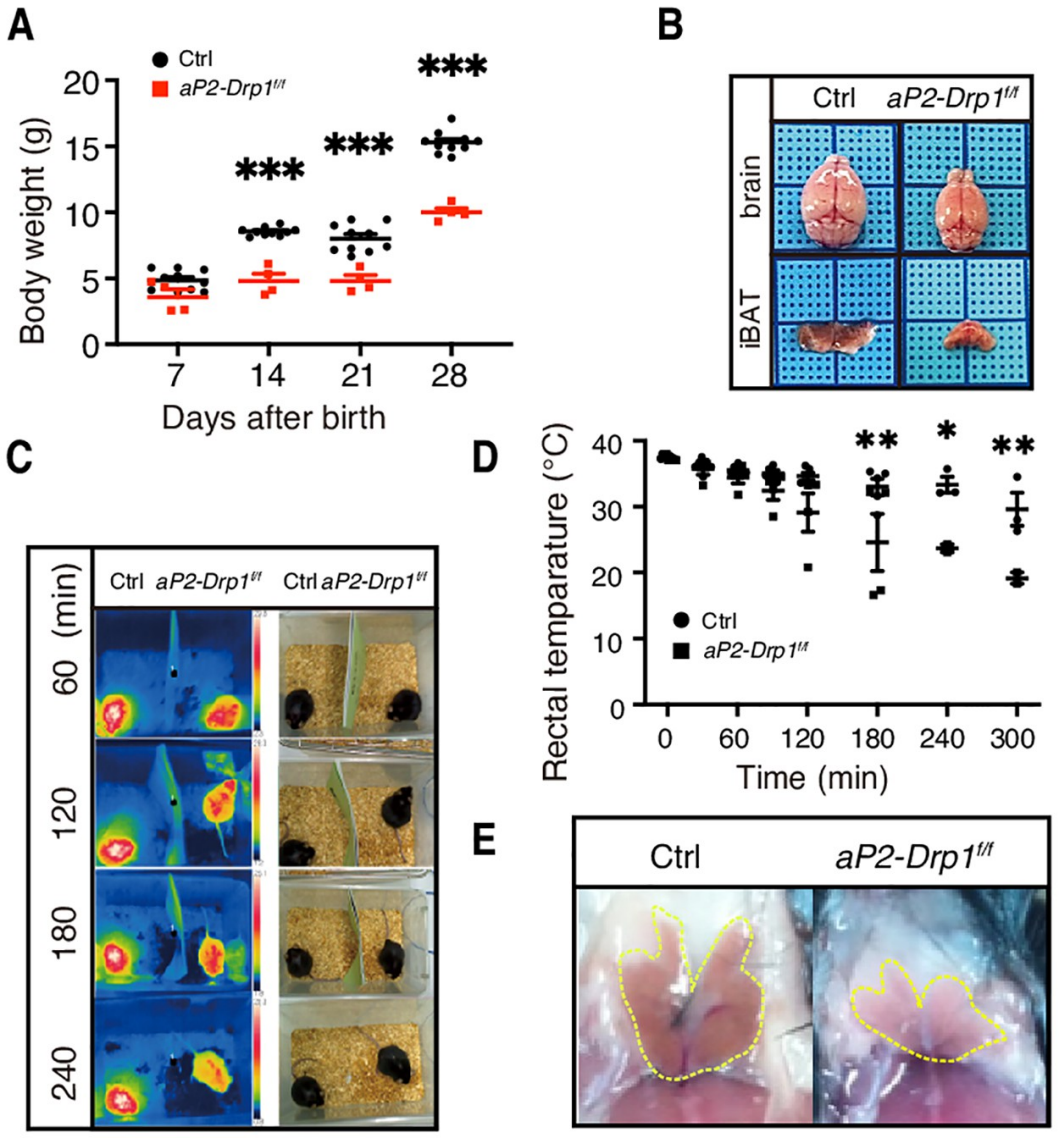
Impaired development of the brain and iBAT of rarely alive adult *aP2-Drp1*^f/f^ mice. A. The body weight of control and *aP2-Drp1*^f/f^ mice on a normal chow diet was measured and is presented as means ± SEM. ***P < 0.001, determined by two-way ANOVA with sidak’s multiple comparisons test. n = 4–10. B. Morphology of the brain and iBAT of control and *aP2-Drp1*^f/f^ mice at 24 weeks after birth. Photographs were taken under the stereoscope. C. Thermographic imaging showing single-cage control and *aP2-Drp1*^f/f^ mouse surface temperature. Mice that were 52 weeks of age were exposed to 4°C temperature for 4 h. Thermografic system FLIR E50 bx shows cooler color according to the temperature at the indicated time. D. Changes in rectal temperature during a cold challenge. *P < 0.05 and **P < 0.01 determined by two-way ANOVA with sidak’s multiple comparisons test (n = 4–5). E. Morphology of iBAT from control and *aP2-Drp1*^f/f^ mice after a cold test. Yellow dashed lines track iBAT boundaries. Abbreviations: Drp1; dynamin-related protein 1, iBAT; interscapular brown adipose tissue.

Only a few *aP2-Drp1*^f/f^ mice (2.4% of total) survived beyond the weaning stage (Table 1). However, these adult *aP2-Drp1*^f/f^ mice remained significantly smaller in size, with body weights markedly reduced compared to age-matched control mice (Fig 5A, Table 3). Morphological analysis was performed on 24-week-old mice. Hypoplasia of the brain and iBAT was observed in adult *aP2-Drp1*^f/f^ mice (Fig 5B). Both the absolute and relative weights of the brain and iBAT were significantly decreased compared to control mice, whereas the absolute and relative weights of the other organs were comparable to those of the control mice, except for the liver relative weight (Table 3). Next, we examined the adult *aP2-Drp1*^f/f^ mice in adaptive thermogenesis *in vivo*. As shown in Fig 5C and 5D, thermographic system FLIR E50bx and rectal temperature measurement revealed a marked drop in temperature within 4 hours after cold challenge to 4°C in *aP2-Drp1*^f/f^ mice, whereas that in control mice remained constant. After the cold challenge test, we observed that the iBAT of *aP2-Drp1*^f/f^ mice were less brownish and smaller compared with the control mice (Fig 5E).

**Table 3:** Comparison of phenotypes between adult control and *aP2-Drp1*^f/f^ mice.

			Control	*aP2-Drp1* ^f/f^	P ^a^
Body weight (g) ^b^			25.88±2.15	17.97±0.34	< 0.05
Organ weight ^b^,^c^	Brain	Absolute	507.43±4.23	241.40±16.06	< 0.001
		Relative	19.87±1.58	13.42±0.80	< 0.05
	iBAT	Absolute	76.67±8.43	24.20±5.10	< 0.01
		Relative	3.01±0.46	1.34±0.28	< 0.05
	liver	Absolute	1073.37±66.51	1023.97±9.16	NS
		Relative	41.64±1.22	57.00±0.95	< 0.001
	spleen	Absolute	137.73±8.08	156.27±17.33	NS
		Relative	5.39±0.52	8.73±1.10	NS
	pancreas	Absolute	127.60±7.91	113.17±9.53	NS
		Relative	4.99±0.45	6.31±0.61	NS
	kidney	Absolute	360.3±14.5	323.63±34.96	NS
		Relative	14.21±1.79	18.06±2.17	NS
	heart	Absolute	168.3±14.05	155.33±21.42	NS
		Relative	6.52±0.33	8.67±1.28	NS
	lung	Absolute	192.37±26.06	164.8±13.61	NS
		Relative	7.39±0.47	9.16±0.71	NS
	eWAT	Absolute	225.57±16.38	176.3±37.51	NS
		Relative	8.74±0.21	9.82±2.14	NS
Litter size ^d^			8.2±1.2	7.5±1.4	NS
Life span (days) ^e^			189.0±10.7	186.7±9.33	NS

^a^, Statistical anaysis was carried out by Student’ s t test, NS: not significant.

^b^, Comparison of absolute (mg) and relative (mg/g body weight) organ weights between 24-week-old mice. n = 3 per group. Values are means ± SEM.

^c^, Wet weights of paired organs were averaged for each mouse, and the single value was used to calculate mean ± SEM among same genotypes.

^d^, Data are mean values derived from six breeding pairs for each genotype.

^e^, Data are mean values of 4 mice (all female) per group.

Abbreviations: iBAT; interscapular brown adipose tissue, eWAT; epididymal white adipose tissue

## Discussion

In this study, we demonstrated that conditional deletion of Drp1 driven by the Fabp4 promoter led to hypoplasia in both BAT and brain. Fabp4, also known as adipocyte-Fabp, is primarily expressed in mature adipocytes and adipose tissues in mammals [23]. Traditionally, the Fabp4 promoter has been widely used to target adipose tissue-specific gene expression in mice. However, it has been shown to have Cre activity in other tissues, including the brain, liver, muscle, adipocyte precursors, and embryonic tissues [24]. The efficiency of Cre recombination is influenced by the target gene locus where loxP sites are located. In adipose tissue, Fabp4 Cre-mediated loxP recombination has been more efficient in BAT than in WAT [25]. In our study, the target gene locus was efficiently deleted in the BAT and brain of newborn mice.

BAT hypoplasia reduced the efficiency of thermogenesis, which may have partly contributed to the postnatal death of *aP2-Drp1*^f/f^ mice. This conclusion is supported by several observations: (i) Fabp4 and Drp1 were localized to the neonatal BAT, and Drp1 protein expression in BAT was significantly decreased in *aP2-Drp1*^f/f^ mice; (ii) most *aP2-Drp1*^f/f^ mice died within 48 hours after birth; (iii) Hotplate support rescued the mice from postnatal death within 12 hours after birth; and (iv) *aP2-Drp1*^f/f^ adult mice were intolerant when exposed to severe cold (4°C). Furthermore, the observation that all surviving mice were female raises questions about the influence of biological sex on metabolic processes and survival outcomes in response to Drp1 knockout. Research on thermogenesis and gender has shown differences in thermogenic capacity between males and females [26–28]. In some animal studies, BAT has been found to be more active in females compared to males. Hormones play a crucial role in modulating thermogenic processes, and gender-specific hormonal profiles contribute to these differences [29, 30]. These hormonal differences between males and females could explain the observed survival differences among littermates in the context of Drp1 knockout, highlighting the importance of considering biological sex in metabolic research.

Thermogenic processes may be classified into shivering and non-shivering phenotypes [31, 32]. Shivering converts the chemical energy of ATP into kinetic energy, causing some of the energy to manifest as heat. Non-shivering thermogenesis primarily depends on BAT. In mice, postnatal thermogenesis is dependent on BAT and is very important for life support [33]. In newborn mice, thermogenesis begins after birth, and the capacity for thermogenesis in BAT increases, accompanied by mitochondrial biogenesis during the first few days [34]. Newborn mice have a large amount of BAT relative to their body weight, indicating that the non-shivering thermogenesis system is essential for maintaining a steady internal temperature and overall health. Human BAT is highly recruited and activated at birth. In contrast, BAT in rats and mice is formed during gestation, making the first few days after birth critical for BAT recruitment in altricial newborns [1, 35]. BAT recruitment in rodents is induced in pups experiencing a cold environment and continues during the first five days after birth, after which it slowly regresses. If animals are born into a thermoneutral environment, postnatal recruitment will be completely inhibited [1]. Previous studies have shown that Drp1 is essential for the early-phase beige adipogenic transcriptional program and plays a crucial role in beige and brown adipogenesis in preadipocytes [12]. Diverging from previous research, our study provides unique insights into Drp1’s contribution to postnatal BAT recruitment.

Stimulation by exposure to a cold environment induces proliferation and differentiation of brown adipocyte precursor cells, hypertrophy of mature brown adipocytes, and the stimulation of Ucp1 activity, expression, and mitochondrial biogenesis [36–38]. Ucp1, mainly expressed in BAT, acts as the primary effector of thermogenesis in rodents and is in the inner mitochondrial membrane. Ucp1 allows the uncoupling of protons moving down their mitochondrial gradient from ATP synthesis, allowing the energy to be dissipated as heat. Ucp1-mediated BAT thermogenesis plays an important role in the maintenance of body temperature and energy balance in rodents [39]. There is a strong correlation between *Ucp1* mRNA expression and environmental temperature. A previous report indicated that inhibition of Ucp1 uncoupling activity decreased Drp1 phosphorylation. Drp1 knock-down impaired Ucp1-dependent mitochondrial uncoupling respiration in brite adipocytes but did not reduce *Ucp1* mRNA and protein levels [40]. Similarly, another study showed that inhibiting of mitochondrial fragmentation by expressing a Drp1 dominant-negative mutant for two days decreased brown adipocytes’ ability to induce mitochondrial uncoupling after activation but did not change Ucp1 expression levels [15]. In *aP2-Drp1*^f/f^ mice, where Drp1 was knocked out using the Cre recombinase system, Drp1 knockout directly affected *Ucp1* expression in BAT, which was not increased under cold temperatures. This suggests that Drp1 is required for both the regulation of key metabolic gene expression and Ucp1-mediated thermogenesis during cold stimulation.

The observed differences in brown adipocyte size and lipid droplet content between *aP2-Drp1*^f/f^ mice and control mice suggest that Drp1 deficiency affects lipid metabolism and storage in brown adipocytes. Specifically, decreased expression of genes involved in *de novo* lipogenesis and lipid droplet formation could lead to reduced lipid accumulation and smaller adipocytes in *aP2-Drp1*^f/f^ mice. Brown adipocytes rely on lipid droplet breakdown and fatty acid oxidation to generate heat during thermogenesis. Therefore, reduced lipid droplet content and altered lipid metabolism in *aP2-Drp1*^f/f^ mice may impair their ability to generate heat in response to cold exposure or other thermogenic stimuli.

Overall, the mechanism by which Drp1 defects induce reduced thermogenesis can be summarized as follows: during cold exposure or noradrenaline treatment, noradrenaline in control mice activates G proteins via β-adrenergic receptors, leading to Drp1-induced mitochondrial fragmentation. This fragmentation facilitates the utilization of fatty acids and enhances thermogenesis [15, 16]. In *aP2- Drp1*^f/f^ mice, several factors contribute to reduced thermogenesis: (i) Brain hypoplasia may impair noradrenaline secretion, reducing the activation of β-adrenergic receptors and subsequent Drp1-induced mitochondrial fragmentation. (ii) The defect in Drp1 directly reduces the expression levels of genes involved in de novo lipogenesis, decreasing lipid droplet content. (iii) The reduced expression of Cpt1 and Ucp1 further diminishes fatty acid oxidation and thermogenesis. Moreover, the modulation of mitochondrial morphology and function by Drp1 highlights potential therapeutic targets for metabolic disorders. By influencing mitochondrial fission and fusion, it may be possible to enhance fatty acid oxidation and energy expenditure, providing new strategies to combat obesity and related metabolic diseases. This dynamic interplay between mitochondrial structure and function emphasizes the critical role of mitochondrial dynamics in energy homeostasis and metabolic regulation. The findings also suggest that interventions aimed at promoting mitochondrial fragmentation could enhance thermogenic capacity and improve metabolic health. Consistent with previous findings, our study implicated Drp1-controlled mitochondrial dynamics as a central mechanism in BAT abundance and activity.

Finally, our findings revealed morphological changes and gene expression alterations. To gain a more comprehensive understanding of the impact of Drp1 knockout on BAT function, additional functional assays, such as direct measurements of thermogenic capacity and mitochondrial respiration assays, are needed. Previous studies have reported that cells expressing a dominant-negative form of Drp1 showed diminished oxygen consumption rates [15]. Future research should focus on elucidating the detailed molecular mechanisms by which Drp1 regulates mitochondrial morphology and function. Understanding these processes could lead to the development of novel therapeutic approaches for metabolic diseases.

## Supporting information

S1 Fig Uncropped western blot images. Uncropped western blot images for Fig 1F (A), Fig 1H (B), and Fig 4C (C).
(PDF)

S2 Fig Morphology of tissues of the *aP2-Drp1*^f/f^ neonatal mice. Tissues from heart, lung, skin, kidney, muscle (leg), and liver of the control and *aP2- Drp1*^f/f^ mice were harvested from neonatal mice and processed for hematoxylin and eosin staining. Scale bar, 50μm.
(PDF)

S1 Table Antibodies used in this study. (PDF)

S2 Table Primers used in this study. (PDF)
