# Supplementary figures and images for: Mitochondrial fission is required for thermogenesis in brown adipose tissue
Source: PLoS One. 2024 Dec 9;19(12):e0312352. doi: 10.1371/journal.pone.0312352 (PMC11627380; doi:10.1371/journal.pone.0312352)

**A**

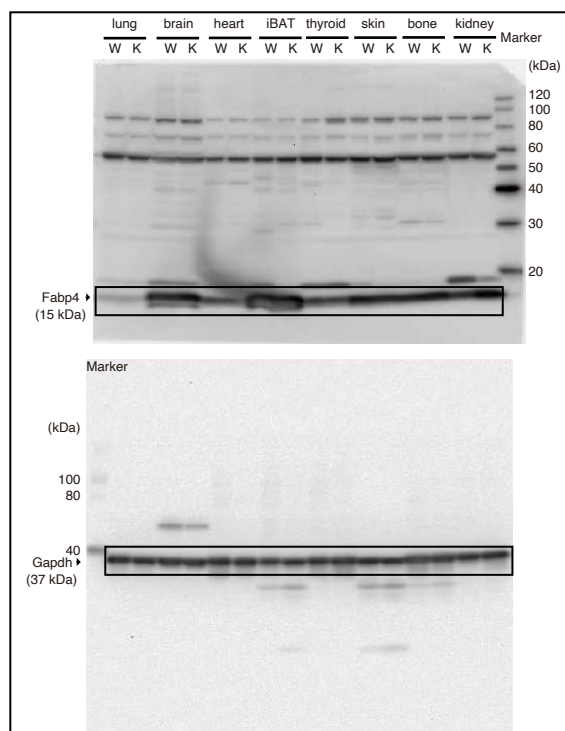

**B**

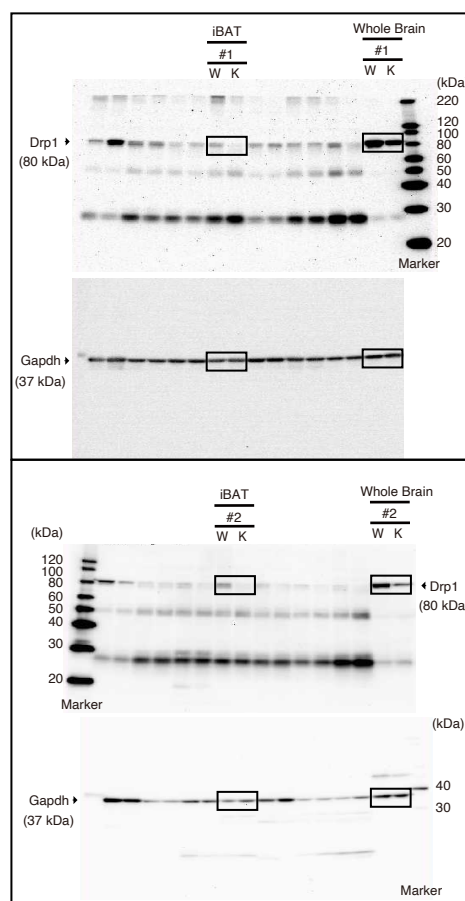

**C**

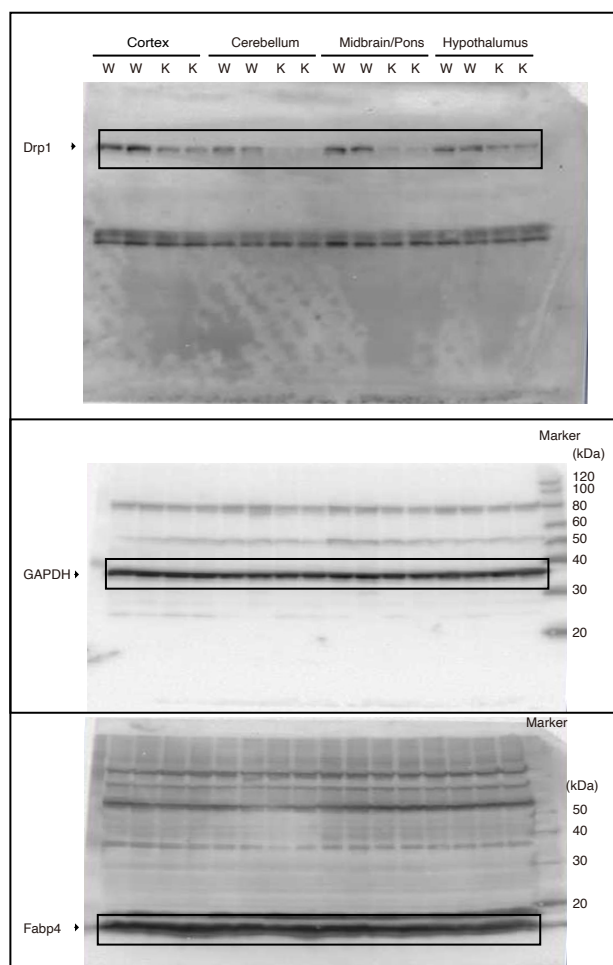

Supplemental Fig. 1 Ibayashi et al.

Supplement: S1 Fig — Uncropped western blot images for Fig 1F (A), Fig 1H (B), and Fig 4C (C). (PDF) [file pone.0312352.s001.pdf]

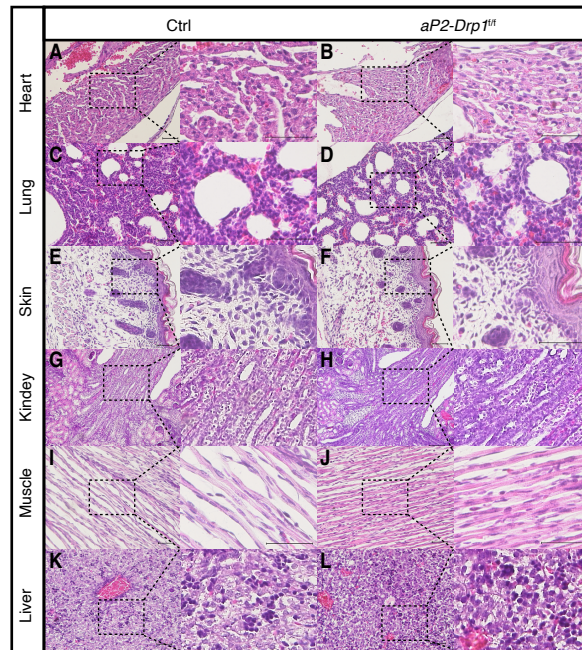

Supplemental Fig. 2 Ibayashi et al.

Supplement: S2 Fig — Tissues from heart, lung, skin, kidney, muscle (leg), and liver of the control and aP2- Drp1f/f mice were harvested from neonatal mice and processed for hematoxylin and eosin staining. Scale bar, 50μm. (PDF) [file pone.0312352.s002.pdf]
