# Supplementary material for: Mitochondrial fission is required for thermogenesis in brown adipose tissue
Source: PLoS One. 2024 Dec 9;19(12):e0312352. doi: 10.1371/journal.pone.0312352 (PMC11627380; doi:10.1371/journal.pone.0312352)
Supplement: S1 Table — (PDF) [file pone.0312352.s003.pdf]

Supplemental Table 1. Antibodies used in this study.

Mouse monoclonal anti-DLP1(DRP1) (1:200 dilution for immunostaining, 1:1000 dilution for western blot ; BD Biosciences, Sparks, MD, USA)
Rabbit anti-FABP4 (1:200 dilution for immunostaining, 1:1000 dilution for western blot; Abcam, Cambridge, UK)
Rabbit monoclonal anti-Tom20 (1:200 dilution; Cell signaling, Danvers, MA)
Rabbit monoclonal anti-GAPDH (HRP Conjugate) (1:5000 dilution; Cell signaling, Danvers, MA)
Horse anti-mouse IgG, HRP-linked antibody (1:5000 dilution; Cell signaling, Danvers, MA, USA)
Goat anti-rabbit IgG, HRP-linked antibody (1:5000 dilution; Cell signaling, Danvers, MA, USA)
Alexa Fluor® 488 donkey anti-rabbit IgG (1:200 dilution; Thermo Fisher Scientific, Rockford, IL, USA)
Alexa Fluor® 594 goat anti-mouse IgG(H+L) (1:200 dilution; Thermo Fisher Scientific, Rockford, IL, USA)
Alexa Fluor® 594 chicken anti-rabbit IgG(H+L) (1:200 dilution; Thermo Fisher Scientific, Rockford, IL, USA)
