# Supplementary material for: Mitochondrial fission is required for thermogenesis in brown adipose tissue
Source: PLoS One. 2024 Dec 9;19(12):e0312352. doi: 10.1371/journal.pone.0312352 (PMC11627380; doi:10.1371/journal.pone.0312352)
Supplement: S2 Table — (PDF) [file pone.0312352.s004.pdf]

Supplemental Table 2. Primers used in this study.

Gene	Forward primer (5' – 3')	Reverse primer (5' – 3')	Product size (bp)
Drp1	TGCCTCAGATCGTCGTAGTG	TGACCACACCAGTTCCTCTG	101
Ucp1	TCTCAGCCGGCTTAATGACT	CCTTCACGACCTCTGTAGGC	66
Cpt1a	CCAGGCTACAGTGGGACATT	GAACTTGCCCATGTCCTTGT	209
Cpt1b	CCCATGTGCTCCTACCAGAT	CCTTGAAGAAGCGACCTTTG	130
Srebp1c	GATCAAAGAGGAGCCAGTGC	TAGATGGTGGCTGCTGAGTG	191
Fas	CTCCGTGGACCTTATCACTA	CTGGGAGAGGTTGTAGTCAG	202
Acc	CCAGGCCATGTTGAGACGCT	ATCACAGAGCGGACGCCATC	132
Cox8b	TGGGGATCTCAGCCATAGTC	TGCTGCGGAGCTCTTTTTAT	89
Prdm16	AGCCCTCGCCCACAACTTGC	TGACCCCCGGCTTCCGTTCA	278
Ppargc1a	CTACAGACACCGCACACACC	GCGCTCTTCAATTGCTTTCT	208
Cidea	GCCGTGTTAAGGAATCTGCTG	TGCTCTTCTGTATCGCCCAGT	113
Gapdh	CCATCACTGCCACCCAGAAG	GATGCAGGGATGATGTTC	91
